# Challenges and responses to infant and young child feeding in rural Rwanda: a qualitative study

**DOI:** 10.1186/s41043-019-0207-z

**Published:** 2019-12-12

**Authors:** Jeanine Ahishakiye, Laura Bouwman, Inge D. Brouwer, Eric Matsiko, Margaret Armar-Klemesu, Maria Koelen

**Affiliations:** 1 Health and Society Chair Group, Wageningen University, Wageningen, The Netherlands; 2 Nutrition and health over the life course chair group, Wageningen University, Wageningen, The Netherlands; 3 Human Nutrition and Dietetics Department, College of Medicine and Health Sciences, University of Rwanda, Kigali, Rwanda; 4 Nutrition Department, Noguchi Memorial Institute for Medical Research, College of Health Sciences, University of Ghana, Legon, Ghana

**Keywords:** Infant and young child feeding, Breastfeeding, Complementary feeding, Qualitative, Rwanda

## Abstract

**Background:**

Despite different interventions to improve child nutrition conditions, chronic malnutrition is still a public health concern in Rwanda, with a high stunting prevalence of 38% among under 5-year-olds children. In Rwanda, only 18% of children aged 6–23 months are fed in accordance with the recommendations for infant and young child feeding practices. The aim of this study was to explore challenges to infant and young child feeding practices and the responses applied to overcome these challenges in Muhanga District, Southern province of Rwanda.

**Methods:**

Sixteen (16) focus group discussions were held with mothers, fathers, grandmothers, and community health workers from 4 rural sectors of Muhanga District. The discussions were recorded, transcribed verbatim, and thematically analyzed using qualitative data analysis software, Atlas.ti.

**Results:**

Two main themes emerged from the data. Firstly, there was a discourse on optimal infant and young child feeding (IYCF) practices that reflects the knowledge and efforts to align with early initiation of breastfeeding, exclusive breastfeeding for the first 6 months, as well as initiation of complementary foods at 6 months recommendations. Secondly, challenging situations against optimal practices and coping responses applied were presented in a discourse on struggling with everyday reality. The challenging situations that emerged as impeding appropriate IYCF practices included perceived lack of breast milk, infant cues, women’s heavy workload, partner relations and living in poverty. Family and social support from community health workers and health facility staff, financial support through casual labor, and mothers saving and lending groups, as well as kitchen gardens, were used to cope with challenges.

**Conclusion:**

Factors influencing IYCF practices are multifaceted. Hence, intervention strategies to improve child nutrition should acknowledge the socially embedded nature of IYCF and address economic and social environmental constraints and opportunities, in addition and above knowledge only.

## Background

Children’s rights to adequate nutrition, good health, and proper development are often violated, especially in developing countries, where undernutrition is one of the leading causes of mortality in children under the age of 5 [1]. The problem is endemic in Sub-Saharan Africa (SSA) and accounts for the highest mortality rate in the world, again especially for under 5-year-olds [2–4]. The causes of undernutrition include, among others, inadequate breastfeeding and complementary feeding practices [5]. Adoption of recommended feeding practices is one of the most effective strategies for optimal nutrition and preventing deaths among children under 5 years of age [6].

In Rwanda, despite the progress achieved in reducing under-5 mortality in the last decades, chronic malnutrition among children continues to be an important public health problem. According to the latest Rwanda demographic and health survey (RDHS), about 38% of under-5-year-old children were stunted in 2015 [7]. Only 30% of breastfed children aged 6–23 months had been fed foods from the minimum number of food groups for their age, 47% were fed the minimum meal frequency, and 18% of Rwandan children aged 6–23 months met the minimum acceptable diet in 2015 [7].

Strategies that aim to improve infant and young child feeding (IYCF) practices have to consider three aspects: *First*, the current emphasis is on feeding practices (breastfeeding and complementary feeding) and its impact on child growth. However, IYCF has a multi-dimensional (food practices, care practices, hygiene-related practices, social network) and multi-level (child, mother–child, household, community, society) nature, and emphasis should be put on the interplay between the dimensions and levels. *Second*, most studies focus on nutritional-physiological aspects such as timing, composition, and frequency of IYCF, not addressing its social embeddedness. IYCF is learned, supported, and expressed in expansive social, everyday-living situations. Hence, the current body of knowledge insufficiently addresses the everyday reality in which IYCF is interwoven with a series of other everyday social practices, influenced by factors operating at different levels. Current strategies therefore may lack relevance and applicability in caregivers’ everyday lives and fail to induce change. *Third*, IYCF is studied merely from a problem orientation, missing out on the responses already applied to overcome challenges. In adverse contexts, there are always people who deploy resources in a specific way that leads to good outcomes. Tapping into existing coping strategies to understand how IYCF operates in different contexts may shed light on context-specific interventions to address malnutrition.

This study considers these aspects by taking the everyday reality in which IYCF is practiced as the point of departure. From here, interrelations between IYCF and other everyday ambitions and practices are investigated. In this first study, the aim was to investigate challenges to infant and young child feeding practices and the responses applied to overcome these challenges in Muhanga District, Southern province of Rwanda.

## Methods

### The study setting

The study was conducted in March 2015 in Muhanga District in Rwanda’s southern province. In 2012, Muhanga District had a population of 318,965 people [8]. Although 39.1% of the Rwandan population was living below the poverty line between 2013 and 2014 [8], Muhanga District was one of the best performers, having reduced the poverty headcount from 53.6% in 2010 to 30.5% three years later. In contrast, the 2014/15 RDHS found that 41.6% of children under the age of 5 were stunted; this is above the national rate of 38% [7]. In 2012, 26% of the households in Muhanga were food insecure against the national average of 21% [9].

### Study population and sampling procedure

**Fig. 1:**
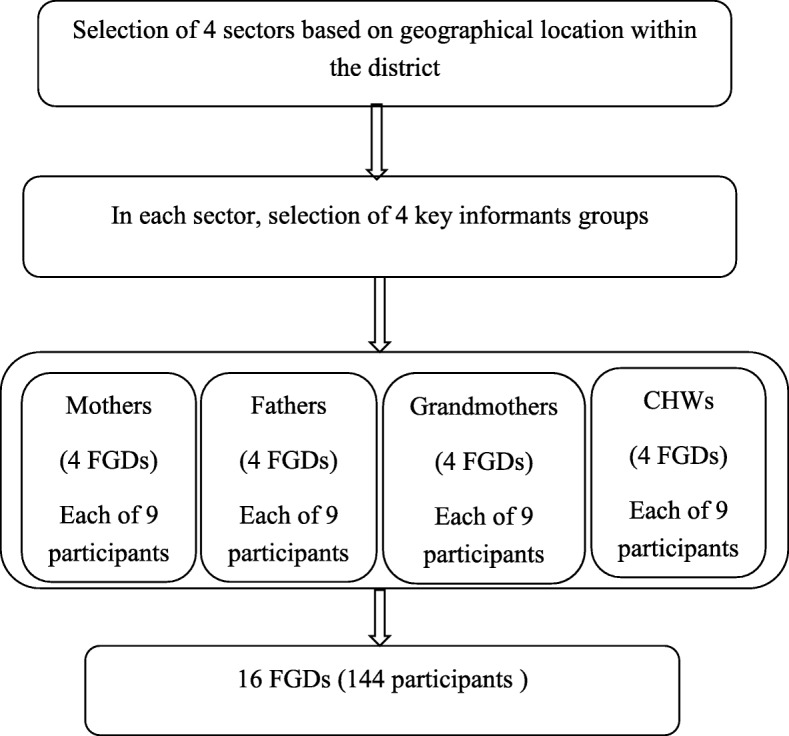
Participants’ sampling procedure Abbreviations: FGDs: focus group discussions; CHWs: community health workers

Data were collected through Focus Group Discussions (FGDs) with four key informant groups: mothers and fathers of infants aged 0–23 months, grandmothers, and community health workers (CHWs). Four administrative sectors were selected using systematic sampling. We assumed that the feeding practices might differ across the district due to the district morphology (variability in landscape). To capture all possible infant and young child feeding practices throughout the district, we purposively selected a sector from the north, a sector from the center, and two sectors from south of the district. In each sector, a purposeful sample per group was selected with the help of the person in charge of community health at health center level and CHWs at the lowest administrative unit (cell). The criteria for selecting key informants were the following: (1) having an infant between 0 and 23 months old for parents; (2) willingness to participate in the study and (3) having personal knowledge and experience in relation to IYCF practices. Separate FGDs were conducted with mothers, fathers, grandmothers, and CHWs. To address age-related variability in child feeding practices, parents were recruited on the basis of four age categories for their children: 0–5 months, 6–8 months, 9–12 months, and 13–23 months. Each age category was represented in FGDs by both mothers and fathers. In each administrative sector, four FGDs were held, each with nine participants from each key informant group, resulting in a total of 144 participants for the entire district (4 key informant groups × 4 FGDs/key informant group × 9 participants/group = 144). The rationale for the number of participants was to capture all possible feeding practices and influences throughout the district. During the fourth FGD in each group, no new information was arising, indicating data saturation, and further inclusion was stopped. Figure 1 summarizes the sampling procedure for participants.

### Data collection

The modules applied by Pelto et al. (2013) in their multi-country focused ethnographic study on child malnutrition in Ghana, South Africa, and Afghanistan [10], as well as in Kenya [11] were adapted and used to guide the FGDs. The discussion guide covered 5 modules on infant and young child feeding: (1) breastfeeding (BF), (2) complementary feeding (CF), (3) food preparation and storage of foods and drinks for infants, and (4) challenges faced by parents of young children, particularly those related to breastfeeding and complementary feeding and responses towards these challenges. Modules on breastfeeding and complementary feeding were in line with WHO indicators for assessing infant and young child feeding practices [12]. The FGDs included open-ended questions and free listing approaches to collect perceptions towards IYCF, perceived challenges, and responses applied to overcome the challenges. Table 1 summarizes the content and focus per module. All FGDs were conducted by a team of two, including the principal investigator as a moderator and a research assistant trained in conducting FGDs as a note-taker. The FGDs were conducted in Kinyarwanda (mother tongue) and each FGD was taking between 40 and 80 min.

**Table 1:** FGD-data collection guide (adapted from Pelto et al., 2013)

Modules	Content	Focus
1. Breastfeeding (BF)	Infants 0–5 months of age - BF initiation, exclusive breastfeeding and other care practices - Difficulties encountered during BF period and coping responses	- Period of exclusive BF (0–5 months) - Community members’ perceptions on BF practices
2. Complementary feeding (CF)	Infants 6–8; 9–12; 13–23 months of age - Timing of introduction of CF - Feeding practices, foods or drinks given to the infants in addition to BF - Difficulties encountered during this period and coping responses	- Community members’ perceptions on CF practices - Care and duties
3. Food preparation, feeding practices, and storage of foods and drinks for infants	Infants 6–8; 9–12; 13–23 months of age - Caregiver behaviors in relation to food preparation and storage	- Specially prepared food for infant (if prepared for whole family, is it modified for the infant, e.g., thinned out?) - Who prepares the food? - How prepared: single portion or extra (if so, which type of storage vessel)? - Who feeds the food to the infant, specifically when mother is away?
4. Breastfeeding and complementary feeding challenges and responses	- Perceptions on challenges, and responses applied to overcome challenges - Community support for IYCF	- Perceptions on breastfeeding and complementary feeding challenges - Responses to challenges - Role of other community members in providing support

### Ethical consideration

The study proposal was reviewed and approved by the Rwanda National Ethics Committee (No 92/RNEC/2015). All the investigators have had research ethics training. Informed written consent was obtained from every participant prior to participation in FGDs. In addition, confidentiality of information obtained was ensured.

### Data analysis

The FGDs were audio-recorded and transcribed verbatim by 2 research assistants. The principal investigator checked the transcripts for quality against the original recordings and against the field notes for accuracy. Atlas.ti analytic software (version 7.5.10) was used for coding and analyzing data. All transcripts were analyzed inductively with respect to the following phases of thematic analysis: familiarization with data, generating initial codes, selection, review, definition, and naming of themes as well as reporting [13]. Codes were reviewed and discussed by the first, second, and third author. These codes in turn were grouped into major families and then into themes representing reported infant feeding practices, challenges, and responses applied to overcome challenges. In presenting the data, relevant verbatim quotes were translated from Kinyarwanda to English by the principal investigator and were reported to aid the interpretation of the data in each theme. Quotations are tagged by participant group (M = mothers, F = fathers, GM = grandmothers, CHW = community health workers) and by sectors of residence (1 = Muhanga, 2 = Kabacuzi, 3 = Nyarusange, 4 = Cyeza).

## Results

### Overview of the results

Two themes emerged from the data: *Firstly*, a discourse on optimal practices that reflects the knowledge about, and efforts to align with recommendations on proper IYCF practices. All aspects were reported by all groups with the exception of affective and responsive breastfeeding, which was not reported by fathers. *Secondly*, challenging situations encountered that hinder optimal practices and responses applied to cope were present in a discourse on struggling with everyday reality (Table 2).

**Table 2:** Overview of results

	Perceptions theme 1: “The way we do it”	Perceptions theme 2: “Struggling with everyday reality”	
IYCF practices	Appropriate IYCF	Challenging situations	Responses
BF practices	- Early initiation of breastfeeding	- Breast milk production is not yet established immediately after birth	- The child is given boiled water to relieve hunger
			- The child is given cow’s milk instead of breast milk for a short period
	- No other foods or drink is given to the baby except breast milk up to 6 months	- The child < 6 months shows appetite	- The food is provided to child
	- Breastfeed the baby on demand	- The mother fears losing occasional daily labor	- The child is not breastfed on demand
	- Importance of mother–child interaction:		
	- Affective and responsive breastfeeding	- Anxiety due to conflict between partners	- Limited care while breastfeeding
	- Touching and eye-to-eye contact with the child while breastfeeding	- Stress due to limited (financial) support from partner	- Limited care while breastfeeding
	- Respect the child’s hunger and satiety cues	- Excessive workload	- Short time for breastfeeding and little mother–child interaction
CF practices	- Introduction of CF at 6 months	- The child older than 6 months refuses food or is not interested	- Continued exclusive BF instead of providing complementary food
	- Foods 6–8 months: porridge, cow’s milk, biscuits, fruits	- Belief that breast milk alone is enough after 6 months of the child’s life	- Continuation of exclusive BF beyond 6 months
	- Foods 9–12 months: beans, sweet potatoes, cooking bananas, vegetables, small fish in addition to porridge, and cow’s milk started earlier	- Excessive workload	- Preparing food from what is available at hand, caring less about the quality
	- Foods 13–23 months: beans, vegetables, sweet potatoes, cassava	- Poverty	- Selling more nutritious and expensive food to buy cheaper food
	- Responsible for preparation: mothers, except when seriously ill (father) or away (female siblings, grandmothers, babysitters)		- Looking for casual labor in the plots of well-to-do neighbors
	- Infant has own pot because of immaturity of digestive system		- Mothers’ saving and lending groups
	- Food stored in closed pot or container and warmed up for next feed		- Kitchen garden

#### Theme 1. “The way we do it”: Discourse on optimal infant and young child feeding practices

This theme represents participants’ discourse on how they attempt to follow recommendations on optimal IYCF:

##### Initiation and exclusive breastfeeding practices

Most of participants across all the different categories of respondents reported that mothers initiate breastfeeding immediately after birth, within the first hour, and that newborn infants do not receive any food or drink immediately after birth except breast milk until they reach 6 months of age. For instance, one father said:

“Giving prelacteal feeds to the new-born no longer exists. The child is fed only breastmilk immediately after birth till 6 months, the time at which complementary foods are introduced.” (F-3)

Participants, across all the different categories, reported that mothers receive information about exclusive breastfeeding immediately after birth from health centers health professionals and community health workers (CHWs).

“Since we are used to giving birth at the health facility, we are taught the advantage of exclusive breastfeeding for the first 6 months. Furthermore once we give birth at the health facility we are all told that it is mandatory to breast feed immediately after birth.” (M-2)

“Even after being discharged and back in the community, the Community Health Workers continue to sensitize mothers to exclusively breastfeed. There is a clear change towards exclusive breast feeding.” (GM-1)

CHWs argued that this awareness resulted from community-based education on the importance of early initiation and exclusive breastfeeding for the first 6 months. As one CHW stated:

“…..mothers used to give hot water to their children immediately after birth, so that they would not cry as breastmilk was not yet established, but thanks to organized community-based campaigns now mothers are aware that the new-born should be given nothing else except breastmilk immediately after birth.” (CHW-1)

Participants discussed not only the practice of exclusive breastfeeding for the first 6 months, but also the way breastfeeding is performed in terms of affection and responsiveness to the child. Most CHWs and mothers were aware of the role of mother–child interaction during breastfeeding episodes, unlike fathers and grandmothers. Participants asserted that mothers pay attention to their babies being breastfed and that breastfeeding is done on demand with respect to the child’s hunger and satiety signals, such as voice and facial expression, as illustrated in the following:

“When breastfeeding, the mother holds well her child close to the breast using her arms and hands to support the baby's head and neck, supports her breasts with her hands and helps the child to latch on in addition to touching and making eye-to-eye contact with the child while breastfeeding, and letting the child himself decide when he has had enough.” (M-1)

“You have to be joyful while breastfeeding. A mother feels delighted when a child smiles at her while being breastfed, both of them feeling that proximity which is a key for motherhood and improved lactation.” **(**CHW-4)

##### Complementary feeding practices

Most of the respondents reported that, besides continued breastfeeding, children receive complementary foods when they are 6 months old and not earlier. The most prominently mentioned food items reported as offered to children between 6 and 8 months are porridge, cow’s milk, biscuits, fruits such as banana and passion fruit. Porridge and cow’s milk are culturally the foods most frequently consumed by infants within the age range of 6 to 8 months in the study area. The porridge is made from one or more of the following cereals: sorghum, maize, soya beans, and wheat cooked with water and sometimes mixed with sugar. Beans, sweet potatoes, cooking bananas, cassava, green leafy vegetables and small fish were most frequently cited by the study participants as foods items consumed by infants aged 9–12 months, in addition to porridge and cow’s milk started earlier. For children within the age range of 13 to 23 months, participants reported that they consume family foods. The findings from the FGDs revealed that generally mothers are the ones responsible for food preparation for infants and young children. It is only during exceptional cases like serious illness of the mother that the father can step in and prepare food. Older female siblings and grandmothers were also cited by participants as helping to make infant and young children’s food when mothers are away.

#### Theme 2. “Struggling with everyday reality”: Challenges impeding optimal IYCF practices and the coping responses applied

Participants talked about various challenging situations inhibiting adequate infant and young child feeding practices as well as the responses used to overcome these challenges.

##### Perceived lack of breast milk

Some mothers believe that breast milk production does not start immediately after delivery. In that context, while waiting for effective breast milk production, boiled water and/or cow’s milk were said to be used as a response to relieve the child’s hunger. For instance, 2 mothers said:

“There is a time when immediately after birth a child gives the impressions that she/he wants to be breastfed but, as a mother believes that breastmilk is not yet produced, she feeds the baby with boiled water, usually using a small spoon.” (M-4)

“Similarly it might happen that there is a mother who does not produce breastmilk for entire 3 days from birth. When a mother squeezes her breast and realize that nothing at all is coming out, in this case it is normal to let the baby have cow’s milk until breastmilk comes in.” (M-1)

##### Infant cues

Although most of participants knew about exclusive breastfeeding for the first 6 months, some participants, across all categories of the respondents, however, revealed that introduction of complementary foods too early before an infant is 6 months old was sometimes practiced. The reasons given for early introduction of complementary foods were that the child showed signs of hunger such as crying as well as showing interest to others eating after breastfed and therefore the need to give food other than breast milk as a response to cope with this.

“It happens that the baby has not reached mature age for eating but he showed an interest in solid food when others are eating. However, considering the hardship a mother goes through to sustain the gains she decides to give food to the baby even before recommended months.” (M-3)

Others mentioned that some mothers delayed to introduce complementary feeding as they believe that breast milk is still enough after 6 months of age, depending on the child’s behavior, such as refusing to eat or showing disinterest in foods. Belief that breast milk is still enough after six months was common for mothers, grandmothers, and fathers.

“It is possible that a child refuses to eat even after 6 months despite repeated attempts at 7 or 8 months not because you didn’t feed him/her but because the child gets enough breastmilk and is less interested in other food.” (M-2)

In this case, participants said that the mothers facing this challenge continue to breastfeed exclusively even beyond six months until she starts noticing the insufficiency of breast milk.

“Sometimes the child is not courageous enough to eat then the mother decides not to pressurize the child at eating but leaves him/her in peace up to 7 or 8 months.” (GM-3)

##### Women’s workload

Participants, mainly mothers, identified heavy workload as a challenging situation for optimal breastfeeding practices where they argue that, because of many household chores and farm work, breastfeeding mothers cannot find enough time to breastfeed their babies. This heavy workload was also found to be responsible for sub-optimal complementary feeding practices by mothers/caregivers. This is illustrated by these quotes:

“Another challenge is the heavy workload for the mother during the exclusive breastfeeding period, where the breastfeeding mother is left with all those household duties which in turn affect the quality of care given to the young child as the mother lacks sufficient time for caring and feeding.” (M-1)

“Inadequate complementary feeding practices are due to excessive workload where mothers spend the day farming and when back home late in the evening she prepares what is available at hand, caring less about the quality.” (M-4)

Participants, mainly mothers and CHWs, mentioned that some mothers breastfeed the baby only when the baby cries and breastfeed concurrently with some other manual work. The latter happens especially for mothers whose livelihood depends largely on occasional daily labor; for instance, during farming activities mothers do not leave their infants at home but carry them along. In some cases, even when the infant is crying for breast milk, the mother does not respond immediately for fear of losing her job. In that condition, participants reported not having access to any response to cope with this situation. As one CHW stated:

“Challenges also include little attention from breastfeeding mothers who only do so upon demand expressed through baby’s crying, while the working woman only breastfeeds her/him to stop crying and then goes back to work immediately.” **(**CHW-4)

Grandmothers, older siblings, and neighbors were mentioned as resources to look after and feed the infant if mothers are away on farm work or paid labor.

##### Partner relations

Limited financial support from a partner at household level was also highlighted by study participants, mainly mothers, as being among the most important challenging situations inhibiting adequate breastfeeding and complementary feeding practices. Participants revealed that some male partners are unsupportive and do not care about contributing to feeding the family and see it as their wives’ business.

“It is only up to a woman to worry about feeding the family and in time of food scarcity she is always more concerned than the husband (male partner) otherwise men are free riders. Those catering for their families living are very few.” (GM-1)

In addition, one mother highlighted the negative effect of family conflicts on the breastfed child.

“Conflicts between partners that lead to anxiety also lead to inadequate care of the child during breastfeeding episodes.” (M-3)

In this situation, the mother reported that they become stressed and loses concentration or pays less attention to breastfeeding the child than she would do in normal circumstances of harmonious family life. Yet, few other mothers and fathers reported appreciable social support from husbands in providing money to buy food, cooking, childcare, and feeding the children.

##### Poverty

Participants, across all the different categories of respondents, linked inadequate breast milk supply to the lack of adequate and sufficient food for mothers due to poverty. In addition, they emphasized that poverty affects complementary feeding practices because poor households do not have enough financial resources to buy food.

“Adequate breastfeeding goes hand in hand with food security. In the absence of the latter, due to poverty, for instance without porridge you cannot aspire to satisfying your baby while breastfeeding.” (M-3)

“Poverty is of course among major constraints for complementary feeding practices. If a mother does not have money then it is obvious she won’t be able to buy milk or cereals for the child.” (CHW-2)

Participants reported a variety of responses to cope with poverty. Some participants said that, in the case of food insecurity caused by poverty, some parents resort to selling more nutritious and expensive foods to buy cheaper ones, and looking for casual manual labor in the plots of their well-to-do neighbors,

“There are those who decide to sell eggs for instance in order to buy potatoes that can be fed and shared among many households members and thus sacrificing nutritious food to acquire alternative bigger quantities food items.”(M-2)

Informal financial support through voluntary mothers’ saving and lending groups as well as growing different kinds of vegetables in their kitchen gardens were used to cope with poverty induced nutrition challenges. For instance, one mother said:

“Women are no longer dependent to their husbands because they formed cooperatives and associations that help them generate revenues and buy nutritious foods without relying on a husband as the breadwinner.” (CHW-2)

“Moreover, here at the community we have been sensitised to have a kitchen garden in each household to ensure good nutrition. We have been trained and we know the nutrition value of having kitchen gardens.”(M-1)

## Discussion

This study investigated challenges to infant and young child feeding practices and the responses applied to overcome these challenges in Muhanga District, Southern province of Rwanda.

Most of the participants were aware of the WHO recommendations of exclusive breastfeeding for the first 6 months. Additionally, participants revealed that most of the mothers in the study community aim to follow the recommended IYCF practices such early initiation of breastfeeding, exclusive breastfeeding for the first 6 months, and timely initiation of complementary foods. Respondents highlighted the importance of responding to infants’ hunger and satiety cues, practicing a warm and affectionate relationship with the child during breastfeeding and complementary feeding. Previous studies show that warm and responsive interactions between caregivers and their children strongly influence children’s health and development [14–16]. Besides the food provided for infants and young children, the way the food is provided to them influences their acceptance of food, dietary intake, and, hence, their growth and development [17, 18].

Poverty was considered by participants as the major challenge affecting exclusive breastfeeding for the first 6 months and complementary feeding practices. Poverty is well known for its negative impact on the proper growth and development of children in Rwanda [19] and elsewhere [20–23]. Despite the damaging effect of poverty, participants reported a variety of responses to overcome poverty-induced nutrition challenges. Participation of mothers in voluntary saving and lending groups was perceived as helping mothers to alleviate poverty, as mothers can borrow and use that money to buy food. This is in line with prior studies highlighting that, when women have more control over the family’s financial resources, a larger proportion of income is allocated for children’s basic needs [24], including food. Therefore, this finding emphasizes the potential of economy-strengthening actions such as mothers’ village savings and loan groups for solving food insecurity induced by poverty. The importance of kitchen gardens in growing different kinds of vegetables and improving dietary diversity was also pointed out by participants. Dietary diversity intervention strategies, including home gardening, have been reported to have a positive influence in overcoming micronutrient deficiencies and thus child undernutrition [6]. Home gardening has also been shown to act as a means of empowering women by enabling them to have greater control over the quality of the family diet [25]. A study conducted in Afghanistan showed that a kitchen garden may improve family food security and access to income for women, who tend to increase spending on children’s health and nutrition [26]. Therefore, there is a need to encourage female involvement in establishing and maintaining kitchen gardens and creating mechanisms for seed supply systems to ensure long-term sustainability.

Women’s laborious work associated with poverty was considered as another challenge for both appropriate breastfeeding and complementary feeding practices among the study participants. Mothers, in addition to being responsible for household chores and farm work, are responsible for childcare and feeding among others. Lack of time to feed and care for the child due to working outside the home including farming activities as well as other household chores has been shown to limit the mother’s ability to use appropriate IYCFP such as exclusive breastfeeding and optimal complementary feeding practices [27, 28]. The challenges that mothers face because of their heavy workload are known to have a negative impact on nutritional outcomes for children [29, 30]. This finding points out the need for interventions to help women allocate more time to caring for and feeding children. Home-based piece work and craft production, including basket weaving, are among the options that could be explored and would replace the more laborious work. Additionally, interventions that focus on providing day-care facilities are also to be encouraged to free mothers during their working hours.

The role of social support in improving IYCF practices is well documented in the literature [31, 32]. The involvement of male partners in breastfeeding promotion and education and providing fathers with knowledge and skills for optimal breastfeeding has been proven to affect exclusive breastfeeding rates positively [33, 34]. In this study, participants reported limited social support from partners for appropriate IYCF practices. The relatively limited male involvement in childcare and child feeding-related activities is very common in non-western societies, including in SSA [31]. Men are in most cases considered as household head, and it is often assumed that they are generally responsible for providing financial and other resources for some household activities including food and for carrying out other tasks critical to family survival [31]. This was not, however, the case in the study area, as participants revealed that some husbands do not care about feeding the family and take it as wives’ business. In such conditions, mothers are restricted in their choice of appropriate IYCF practices without financial support from their husbands. Consequently, providing mothers with adequate nutrition knowledge and education on appropriate IYCF practices has little influence without the involvement of their husbands or partners as the financial gatekeepers [35]. Intervention programs should consider ways to increase the engagement of men in child feeding and caregiving.

Despite the role commonly ascribed to grandmothers as the guardians of tradition [36], social support from grandmothers may have a positive effect on child feeding practices. Grandmothers in the study area were also said to help mothers in child feeding and caring. This finding is similar to other studies in different settings [31, 36–38]. A study conducted in Kenya showed that encouraging the provision of social support to mothers by key household influencers such as grandmothers and fathers improved some targeted infant feeding practices, such as feeding the infant the minimum number of meals and dietary diversity [32]. These promising results support the need to adopt a wider, family-centered approach by providing resources such as more education to these influential family members to enhance support in child health, especially in optimal IYCF and care practices, as these relatives have less access to new knowledge than mothers do.

The strength of this study is its respondent’s diversity. The ideas voiced out can therefore be taken as an exact reflection of community knowledge, beliefs, and practices. Nevertheless, the study suffered from a number of limitations: Firstly, the participants were recruited in only one district, Muhanga, the findings may not be generalized to populations outside this area due to some specificities as well as the less representative sample from one District. However, as data saturation was reached during data collection, the findings were adequate to provide a deeper understanding of challenges and responses to infant feeding practices that allow for a judgment of the extent to which findings can be relevant and applicable to other similar settings. Secondly, there was inability to observe the actual infant and young child feeding practices and behaviors as were reported by participants during the focus group discussions. Future research that confirms self-reported interview data with direct observations of IYCF practices in everyday life would be valuable. Thirdly, participants might have over-reported the practices and influences due to social desirability. This might have been more evident for the grandmothers’ responses as grandmothers’ advice and concerns may reflect cultural beliefs and infant feeding practices that do not protect appropriate IYCF. However, the interviewers asked the same questions in different forms as much as possible to check for consistency in the responses.

## Conclusion

This study finds that appropriate IYCF is not only about food practices—paying attention to the quality, diversity, and amount of food being offered to children—but also about caregivers’ responsiveness and affection during feeding episodes. The study reveals that a number of challenges including living in poverty, women’s heavy workload, limited financial support from partners, and seasonal fluctuations in food availability are perceived as impeding appropriate IYCF. Family and social support from CHWs and health facility staff, financial support through casual labor, mothers’ saving and lending groups, and kitchen gardens were used to overcome challenges. Factors influencing IYCF practices are multifaceted. Hence, intervention strategies to improve child nutrition should acknowledge the socially embedded nature of IYCF and address economic, social environmental constraints and opportunities in addition and above knowledge only.

## Data Availability

The data generated and analyzed during the current study are available from the corresponding author on reasonable request**.**

## Abbreviations

BF: Breastfeeding
CF: Complementary feeding
CHWs: Community health workers
FGD: Focus group discussion
IYCF: Infant and young child feeding
NISR: National Institute of Statistics Rwanda
RDHS: Rwanda Demographic and Health Survey
RNEC: Rwanda National Ethics Committee
SSA: Sub-Saharan Africa
WHO: World Health Organization
